# Association of active/passive smoking and urinary 1-hydroxypyrene with poor sleep quality: A cross-sectional survey among Chinese male enterprise workers

**DOI:** 10.18332/tid/90004

**Published:** 2018-05-22

**Authors:** Bo Zhou, Yifei Ma, Fu Wei, Li’e Zhang, Xiaohong Chen, Suwan Peng, Feng Xiong, Xiaowu Peng, Bushra NiZam, Yunfeng Zou, Kaiyong Huang

**Affiliations:** 1 Research Center for Regenerative Medicine, Guangxi Medical University, Nanning, China; 2 Department of Toxicology, School of Public Health, Guangxi Medical University, Nanning, China; 3 AIDS Prevention and Control Institute, Liuzhou Center for Disease Control and Prevention, Liuzhou, China; 4 Department of Physical Examination, Guangxi Institute of Occupational Disease Prevention and Treatment, Nanning, China; 5 Center for Environmental Health Research, South China Institute of Environmental Sciences, Ministry of Environmental Protection, Guangzhou, China; 6 Department of Occupational Health and Environmental Health, School of Public Health, Guangxi Medical University, Nanning, China

**Keywords:** sleep quality, active smoking, passive smoking, 1-hydroxypyrene

## Abstract

**INTRODUCTION:**

Tobacco use has been implicated as an important factor for poor sleep quality. However, in most studies, the sleep quality of smokers was only assessed though a self-reported questionnaire, without measuring any internal biomarkers that reflect the levels of tobacco exposure. We examined the association of active and passive smoking with sleep quality, assessed smoking exposure using urinary 1-hydroxypyrene (1-HOP) as an internal biomarker, and further explored the relationship between 1-HOP and sleep quality.

**METHODS:**

A cross-sectional survey was conducted in Liuzhou city, Guangxi, China. A total of 1787 male enterprise workers were enrolled. The smoking attribute data were collected by self-reported questionnaire, and individual sleep quality was evaluated through the Pittsburgh Sleep Quality Index (PSQI). The concentration of urinary 1-HOP was measured by high-performance liquid chromatography.

**RESULTS:**

Compared with non-smoking, active smoking and passive smoking were significantly associated with long sleep latency (odds ratio, OR=1.84, 95% confidence interval, CI=1.28–2.64; 1.45, 1.00–2.11, respectively), short sleep duration (OR=2.72, 95% CI=1.45–5.09; 1.94, 1.01–3.71, respectively), daytime dysfunction (OR=1.54, 95% CI=1.10–2.17; 1.44, 1.02–2.03, respectively), and overall poor sleep quality with PSQI total score >5 (OR=1.41, 95% CI=1.05–1.88; 1.34, 1.00–1.79, respectively). Compared with non-smokers, active smokers had higher urinary 1-OHP concentrations that were significant (p=0.004), while passive smokers had no significant difference in urinary 1-OHP concentration (p=0.344). The high concentration group was significantly associated with daytime dysfunction and overall poor sleep quality with PSQI total score >5 (OR = 1.73, 95% CI=1.06–2.81; 1.76, 1.18–2.63, respectively).

**CONCLUSIONS:**

Both active smoking and passive smoking are risk factors for poor sleep quality among Chinese male enterprise workers. Active smokers had significantly higher levels of urinary 1-OHP than non-smokers, and high concentration of 1-OHP was associated with daytime dysfunction and overall poor sleep quality.

## INTRODUCTION

Poor sleep quality is one of the most commonly reported health problems, which links to increased risk of depression, anxiety, low work efficiency, motor vehicle or occupational accidents, obesity, cardiovascular disease, and diabetes^1-3^. Epidemiological surveys indicate that about 26–35% of adults have poor sleep quality^4-6^, which is associated with increased mortality among men^7,8^. A number of studies have shown that sleep quality is affected by many factors, including smoking^6,9^, drug and alcohol abuse^10^, and social support^11^.

The association of active smoking with poor sleep quality has been well documented in previous studies, indicating that the smokers, compared with non-smokers, were more likely to experience poor sleep quality, such as short sleep duration, long sleep latency, early morning awakening, sleep disturbances, and habitual snoring^6,10,12,13^. However, some other studies reported that there is no relationship between smoking and long sleep latency, short sleep duration and early morning awakening^14,15^. One study even indicated contrary results, showing that cigarette use could reduce individual sleeping problems, such as trouble staying asleep and waking often in the night^16^. It is noteworthy that most of these studies did not take passive smoking into consideration. The influence of passive smoking on sleep quality might provide comprehensive understanding of the association of smoking with sleep quality. In recent years, the effects of passive smoking, or secondhand smoke (SHS) exposure, on sleep have attracted increasing attention. Some studies have identified that, compared with non-smoking, passive smoking was significantly associated with short sleep duration, subjective insufficient sleep, difficulty in initiating sleep, restless sleep, and snoring loudly^17-20^. However, these studies examining the association of passive smoking with sleep problems mainly focused on pregnant women, children or adolescents, and few studies paid attention to worker population.

In addition, most studies in the field assessed sleep quality of active smokers or passive smokers only through a self-reported questionnaire, without measuring any internal biomarkers to reflect tobacco smoking exposure or including some influencing factors, such as physical exercise, siesta, working hours and family concern, which might lead to biased results. A cross-sectional study conducted in Japan used the Japanese version of the generic NIOSH (National Institute for Occupational Safety and Health) job-stress questionnaire to examine the association of active and passive smoking with sleep disturbances and found that men who were exposed to passive smoking at work were associated with short sleep duration6. In this study, we examined the association between both active and passive smoking with sleep quality using the Pittsburgh Sleep Quality Index (PSQI) and assessed the levels of tobacco smoking exposure by measuring urinary 1-hydroxypyrene (1-HOP), an internal biomarker.

Polycyclic aromatic hydrocarbons (PAHs) are a regarded as important organic pollutants resulting from incomplete combustion of fossil fuels in gasoline, burning coal, diesel-powered vehicles, domestic air heating, cooking and tobacco smoking^21-23^. The internal biomarker 1-OHP, the most common metabolite of PAHs, has been recommended as being dependable in evaluating recent exposure to PAHs^21,24,25^. Previous studies have shown that the levels of urinary 1-OHP in smokers, or in SHS exposed populations, were significantly higher than those in non-smokers or in people unexposed to SHS, respectively^21,26^. In the present study, urinary 1-HOP was used as an internal biomarker to assess the levels of tobacco smoke exposure.

The aim of this cross-sectional study is to examine the association of active and passive smoking with sleep quality among Chinese male enterprise workers and to assess tobacco smoking exposure by measuring urinary 1-HOP and the relationship between urinary 1-HOP and sleep quality.

## METHODS

### Study design and study population

This is a cross-sectional study started in August 2014. The study population came from a prospective cohort study for employees working in a machinery company in Liuzhou city, Guangxi, China. We recruited 1906 male workers for this study. All the respondents were asked to fill in a questionnaire including sociodemographic characteristics, personal mental health, family concern and sleep quality assessment^27^. The questionnaire was distributed by investigators who were trained in advance. Once a questionnaire was completed it was checked on-the-spot by the investigators. For any unfinished questions or illogical answers, investigators reconfirmed the answers through face-to-face interviews. After double checking, we excluded 119 participants either for missing information in the questionnaires or being diagnosed to have diseases that might affect the outcome (e.g. serious chronic diseases, nervous system diseases)^27^. Finally, a total of 1787 respondents were included in the study.

### Sociodemographic variables

We collected sociodemographic variables as follows: age; marital status (single, married, divorced); educational level (primary school, junior high school, high school, college or university); body mass index (BMI, calculated as weight [kg] divided by height [m] squared; BMI ≥ 28 is defined as ‘obese’, BMI 24.0–27.9 ‘overweight’, BMI 18.5–23.9 ‘normal’, and BMI < 18.5 ‘underweight’)^28^; drinking status (current, previous, or never); smoking status (current, previous, or never); passive smoking (yes or no; ‘passive smoking’ for non-smokers was defined for those in close contact with a smoker for >1 hour/week); physical exercise (yes or no; ‘physical exercise’ was defined as doing any physical sport >3 times/week and >15 minutes each time); siesta (yes or no; ‘siesta’ was defined as having a nap 12–3 p.m. more than 4 times per week and >15 minutes each time); shift work (yes or no); working hours (per day); working days (per week); and physical labor intensity grading (light, middle, heavy).

### Assessment of personal mental health and family concern

The 12-item version of the General Health Questionnaire (GHQ-12), widely used in screening instruments for affective disorders, was used to assess personal mental health in our study. The GHQ-12 has six positive and six negative questions with response choices including: less than usual, no more than usual, more than usual and much more than usual^29^. The first two choices scored 0, and the next two scored 1. The total score for each respondent ranged from 0 to 12, with the higher scores indicating higher mental distress. Those who scored less than 3 were defined as having good mental health, while those who scored ≥3 on the GHQ-12 scale were defined as having a mental disorder.

The Family Concern Index questionnaire was used to assess respondents’ satisfaction with family support. It consists of 5 items: adaptability, partnership, growth, affection, and resolution, with a 3-point response scale ranging from 0 (hardly ever) to 2 (almost always) to reflect the frequency of feeling satisfied^30,31^. The total score of five items ranged from 0 to 10, with the higher score suggesting better family functioning. Score ranges 7–10, 4–6, 0–3 indicate positive family function, moderate family dysfunction, severe family dysfunction, respectively.

### Assessment of sleep quality

The Pittsburgh Sleep Quality Index (PSQI) was employed to evaluate participants’ quality of sleep^32^. This validated scale evaluates multiple dimensions of sleep during a 1-month period and consists of seven components: sleep duration, sleep latency, sleep disturbances, subjective sleep quality, habitual sleep efficiency, daytime dysfunction, and use of sleeping medication. The score of each component ranges from 0 to 3. The final score of sleep quality is the sum of the seven scores, ranging from 0 to 21, with the higher total score suggesting poorer sleep quality^32^. A PSQI total score of > 5 indicates poor sleep quality.

### Measurement of urinary 1-hydroxypyrene (1-HOP)

All individuals were asked to provide a sample of spot urine (20 mL) before having breakfast. Regrettably, 1226 respondents could not offer the urine sample on schedule. Finally, we collected 561 valid urine samples from 172 active smokers, 138 non-smokers and 251 passive smokers. The concentration of 1-OHP was determined by high-performance liquid chromatography (HPLC), as described previously^33^. Urinary 1-OHP concentration was adjusted by urinary creatinine excretion (μmol/mol creatinine). Referring to the kit instructions supplied by the Nanjing Jiancheng Biological Engineering Institute, urinary creatinine was assessed by ammonia iminohydrolase-PAP^27^. Urinary 1-HOP content was divided into three groups (low concentration <0.76 μmol/mol, middle concentration 0.76–1.52 μmol/mol and high concentration >1.52 μmol/mol) according to tri-sectional quantiles^34^.

### Statistical analysis

Statistical analysis was performed using SPSS (Version 19.0, SPSS Inc., Chicago, Illinois, USA). Mean and standard deviation (SD) were used for continuous variables, while frequency and percentage were used to describe categorical variables. The comparisons of categorical covariates among the different smoke exposure groups were performed using chi-squared test and continuous measures using analysis of variance or Mann-Whitney U test when the data were not conformed to normal distributions. Multivariate logistic regression model was used to estimate the relationship between the PSQI components and different smoke exposure groups, after adjusting for age, marital status, educational level, BMI, drinking status, physical exercise, siesta, shift work, working hours, working days, mental health, and family function. Mann-Whitney U test was also employed to compare the concentration of urinary 1-HOP among different smoke exposure groups. We then used another multivariate logistic regression model to assess the association between PSQI components and urinary 1-HOP levels. A p-value of <0.05 was considered statistically significant.

## RESULTS

### Sociodemographic characteristics of study population

Totally 1787 male workers were recruited in our study with the mean age of 35.37 ± 7.77 years (mean ± SD). Of the respondents with ‘educational level as primary and junior high school’, ‘currently drinking’, and ‘previous drinking’, over 60% were active smokers, and 56% of the workers who did not do physical exercise were active smokers. The number of active smoking respondents with ‘no shift work’ or ‘getting use to siesta’ were significantly less than those with ‘shift work’ or ‘no siesta’, respectively (50% vs 57%, p<0.01 or 50% vs 65%, p<0.01, respectively). There was no significant difference in the frequency of age groups, marital status, BMI, working hours, working days, physical labor intensity grading, mental disorder, and family concern among non-smokers, active smokers, and passive smokers (Table 1).

**Table 1:** Sociodemographic characteristics of the study population for the different tobacco smoke exposure groups

	*Total (N=1787)*	*Non-smoking (N=307)*	*Passive smoking (N=562)*	*Active smoking (N=918)*	

*Characteristics*	*N (%)*	*N (%)*	*N (%)*	*N (%)*	*p*
**Age (years) (mean ± SD)**	35.37±7.77	35.73±7.92	34.56±7.58	35.74±7.81	0.012
**Age group**					0.177
18–25 years	160 (9)	26 (16, 8)	59 (37, 10)	75 (47, 8)	
26–29 years	418 (23)	75 (18, 25)	138 (33, 25)	205 (49, 22)	
30–39 years	625 (35)	96 (15, 31)	204 (33, 36)	325 (52, 36)	
≥ 40 years	584 (33)	110 (19, 36)	161 (27, 29)	313 (54, 34)	
**Marital status**					0.079
married	1272 (71)	222 (17, 72)	384 (30, 68)	666 (53, 73)	
single	463 (26)	78 (17, 26)	166 (36, 30)	219 (47, 24)	
divorced	52 (3)	7 (13, 2)	12 (23, 2)	52 (64, 3)	
**Educational level**					<0.001
primary and junior high school	389 (22)	52 (13, 17)	93 (24, 17)	244 (63, 27)	
high school	928 (52)	156 (17, 51)	296 (32, 53)	476 (51, 52)	
college or university	470 (26)	99 (21, 32)	173 (37, 30)	198 (42, 21)	
**Drinking status**					<0.001
current	1037 (58)	121 (12, 39)	248 (24, 44)	668 (64, 73)	
previous	57 (3)	6 (11, 2)	15 (26, 3)	36 (63, 4)	
never	687 (39)	180 (26, 59)	297 (43, 53)	210 (31, 23)	
**Physical exercise**					0.003
yes	1109 (62)	211 (19, 69)	361 (33, 64)	537 (48, 58)	
no	678 (38)	96 (14, 31)	201 (30, 36)	381 (56, 42)	
**BMI**					0.070
underweight	106 (6)	11 (10, 4)	33 (31, 6)	62 (59, 7)	
normal	982 (55)	161 (16, 52)	303 (31, 54)	518 (53, 56)	
overweight	566 (32)	117 (21, 38)	181 (32, 32)	268 (47, 29)	
obese	130 (7)	18 (14, 6)	44 (34, 8)	68 (52, 8)	
**Siesta**					<0.001
yes	1534 (86)	268 (17, 87)	511 (33, 91)	755 (50, 82)	
no	253 (14)	39 (15, 13)	51 (20, 9)	163 (65, 18)	
**Shift work**					0.008
yes	423 (24)	71 (17, 23)	109 (26, 19)	243 (57, 26)	
no	1364 (76)	236 (17, 77)	453 (33, 81)	675 (50, 74)	
**Working hours per week**					0.491
< 8 hours	85 (5)	12 (14, 4)	26 (31, 5)	47 (55, 5)	
8 hours	1559 (87)	266 (17, 87)	486 (31, 86)	807 (52, 88)	
> 8 hours	143 (8)	29 (20, 9)	50 (35, 9)	64 (45, 7)	
**Working days per week**					0.991
< 5 days	63 (3)	10 (16, 3)	20 (32, 4)	33 (52, 4)	
5 days	1123 (63)	195 (17, 64)	356 (32, 63)	572 (51, 62)	
> 5 days	601 (34)	102 (17, 33)	186 (31, 33)	313 (52, 34)	
**Physical labor intensity grading**					0.728
light	54 (3)	10 (18, 3)	15 (28, 3)	29 (54, 3)	
middle	1448 (81)	253 (17, 83)	462 (32, 82)	733 (51, 80)	
heavy	285 (16)	44 (15, 14)	85 (30, 15)	156 (55, 17)	
**Mental disorder**					0.259
yes	211 (12)	29 (14, 9)	64 (30, 11)	118 (56, 13)	
no	1576 (88)	278 (18, 91)	498 (32, 89)	800 (50, 87)	
**Family concern**					0.386
Positive family function	1126 (62)	182 (16, 59)	362 (32, 64)	582 (52, 63)	
Moderate family dysfunction	525 (30)	101 (19, 33)	164 (31, 29)	260 (50, 28)	
Severe family dysfunction	136 (8)	24 (18, 8)	36 (26, 7)	76 (56, 9)	

(x, y): x was calculated by rows, and y was calculated by columns.

### Association between sleep quality and tobacco smoke exposure status

Multivariate logistic regression analysis indicates that, compared with the non-smoking (reference group), active smoking and passive smoking were significantly associated with long sleep latency, short sleep duration, daytime dysfunction, and overall poor sleep quality with PSQI total score >5. There was no significant difference in subjective sleep quality, habitual sleep efficiency, sleep disturbances, and use of sleeping medication among different groups. More details are shown in Table 2.

**Table 2:** Adjusted odds ratios for sleep quality for the different tobacco smoke exposure groups

*Sleep Variable*	*Non-smoking (N=307)*	*Passive smoking (N=562) OR ( 95% CI)*	*Active smoking (N=918) OR ( 95% CI)*
Subjective sleep quality ≥2	1 (reference)	1.30 (0.82–2.04)	1.40 (0.89–2.14)
Sleep latency ≥2	1 (reference)	1.45 (1.00–2.11)	1.84 (1.28–2.64)
Sleep duration ≥2	1 (reference)	1.94 (1.01–3.71)	2.72 (1.45–5.09)
Habitual sleep efficiency ≥2	1 (reference)	1.52 (0.83–2.78)	1.34 (0.74–2.43)
Sleep disturbances ≥2	1 (reference)	1.20 (0.74–1.95)	1.03 (0.64–1.66)
Use of sleeping medication ≥2	1 (reference)	2.79 (0.29–26.67)	3.13 (0.37–26.77)
Daytime dysfunction ≥2	1 (reference)	1.44 (1.02–2.03)	1.54 (1.10–2.17)
PSQI total score >5	1 (reference)	1.34 (1.00–1.79)	1.41 (1.05–1.88)

Model was adjusted for age, marital status, educational level, BMI, drinking status, physical exercise, siesta, shift work, working hours, working days, mental health, and family function. OR: odds ratio, CI: confidence interval.

### Urinary 1-OHP concentration and its relationship with sleep quality

Compared with the non-smoking group, the active smoking group had higher urinary 1-OHP concentration (adjusted by urinary creatinine) that was significant (p=0.004) (Table 3), however, passive smoking had no significant difference in urinary 1-OHP concentration (p=0.344). In addition, the urinary 1-OHP concentration in active smokers was significantly higher than that in passive smokers (Table 3).

**Table 3:** Comparison of urinary 1-OHP concentration (median) among the different tobacco smoke exposure groups

	*Variable*	*Non-smoking (N=138)*	*Passive smoking (N=251)*	*Active smoking (N=172)*
	Urinary 1-OHP concentration (adjusted by urinary creatinine, μmol/mol)	0.97	1.02#	1.35*§

Compared to non-smoking:

# p=0.344

* p=0.004;

compared to passive smoker:

§ p=0.010.

Taking the low concentration of urinary 1-OHP (<0.76 μmol/mol) as a reference group, the high concentration group was significantly associated with daytime dysfunction and overall poor sleep quality with PSQI total score >5. However, there was no association between the other sleep components and urinary 1-HOP level (Table 4).

**Table 4:** Odds ratios for sleep quality among different urinary 1-OHP levels

*Sleep Variable*	*Low concentration (N=191)*	*Middle concentration (N=181) OR ( 95% CI)*	*High concentration (N=189) OR ( 95% CI)*
Subjective sleep quality ≥2	1 (reference)	1.90 (0.92–3.92)	1.58 (0.85–2.95)
Sleep latency ≥2	1 (reference)	1.06 (0.58–1.94)	1.11 (0.64–1.92)
Sleep duration ≥2	1 (reference)	0.77 (0.29–2.06)	0.61 (0.26–1.44)
Habitual sleep efficiency ≥2	1 (reference)	0.78 (0.33–1.83)	1.09 (0.48–2.48)
Sleep disturbances ≥2	1 (reference)	1.51 (0.70–3.24)	1.33 (0.67–2.64)
Use of sleeping medication ≥2	1 (reference)	0.79 (0.04–14.38)	0.50 (0.04–6.48)
Daytime dysfunction ≥2	1 (reference)	0.97 (0.57–1.64)	1.73 (1.06–2.81)
PSQI total score >5	1 (reference)	1.42 (0.91–2.23)	1.76 (1.18–2.63)

Low 1-OHP concentration: < 0.76 μmol/mol, middle 1-OHP concentration: 0.76–1.52 μmol/mol, high 1-OHP concentration: >1.52 μmol/mol.

Model adjusted for age, marital status, educational level, BMI, drinking status, physical exercise, siesta, shift work, working hours, working days, mental health, and family function. Abbreviations: OR: odds ratio, CI: confidence interval.

## DISCUSSION

To our knowledge, this is the first study examining the association of active and passive smoking with sleep quality among Chinese male enterprise workers using the Pittsburgh Sleep Quality Index (PSQI), and simultaneously assessing tobacco smoking exposure by measuring urinary 1-HOP as an internal biomarker. This study revealed that both active smoking and passive smoking were significantly associated with certain PSQI components and overall poor sleep quality with PSQI total score >5. Additionally, we also found that the urinary 1-OHP concentration in active smokers was highest, followed by passive smokers and non-smokers, and high concentration of urinary 1-OHP was significantly associated with overall poor sleep quality.

Some previous population-based cross-sectional studies reported that active smoking was significantly associated with poor sleep quality, such as long sleep latency, short sleep duration, subjective lower sleep efficiency, sleep disturbances, subjective insufficient sleep, difficulty awakening in the morning, and early morning awakening^20,35^. A number of studies have indicated that many other factors (e.g. age, education, obesity, alcohol use, exercise, mental health) were related to sleep quality too. A general population-based study in China found that old age, female, divorced or low level of education were risk factors for all types of insomnia^36^. Physical exercise had significant positive correlation with increased sleep quality^37^, while obesity and heavy drinking was significantly associated with frequent sleep insufficiency^5^. After adjusting for age, marital status, educational level, BMI, drinking status, physical exercise, siesta, shift work, working hours, working days, mental health, and family function in the multivariate logistic regression analysis model, our data showed that increased risk of long sleep latency, short sleep duration, daytime dysfunction, and overall poor sleep quality with PSQI total score >5 were found in active smokers, compared with the non-smokers, but no relationship was found between active smoking and other sleep components. Similar results were found in some recent studies^38-40^. Because of the stimulant effect of nicotine, a smoker’s brain had enhanced/maintained alertness, which led to difficulty initiating sleep and prolonged sleep latency^41,42^. Previous studies reported that smokers were more likely to report early morning awakening, resulting in reduced sleep duration and short total sleep time during the night^6,40^. The possible reason for association of smoking with daytime dysfunction might be that poor sleep quality during the night interrupts restoration of fatigue and makes people feel tired and confused^12,27^.

However, a similar study conducted in Japan among 2628 workers from 300 factories reported no association between current smoking with difficulty of initiating sleep, difficulty of maintaining sleep, and sleep duration among male workers^6^. Furthermore, a recent study conducted in Chinese adolescents showed that cigarette smoking was significantly associated with hypnotic medication use^10^, but an inconsistent result was observed in our study. Several explanations are possible for these different results: 1) the sample size and/or composition of the study population were/was significantly different, 2) methods for assessing sleep quality were different, 3) in some studies, potential confounders (such as working days per week, shift work, mental illness, stress, caffeine intake) were not measured^1,40^, and 4) some influencing factors (such as physical exercise, siesta, working hours, physical labor intensity, and family concern) were not taken into account^6^. In order to better evaluate the association between sleep quality and smoking, further research should include various potential confounding factors and examine relevant internal exposure markers.

Although the exact mechanism of passive smoking affecting sleep quality was not certain, our study found that passive smoking was significantly associated with long sleep latency, short sleep duration, and daytime dysfunction in male enterprise workers. Cross-sectional studies in Japan reported similar results, i.e. that passive smokers were more likely to report short sleep duration, compared to non-smokers without SHS exposure^6,18^. A possible explanation for this finding is that passive smoking might irritate the upper airways inducing uncomfortable manifestations leading to long sleep latency and short sleep duration. We found that passive smoking was not associated with sleep disturbances, as found in a study based on the data of the National Health and Nutrition Examination Survey in the USA, 2005–2006^1^.

The internal biomarker, 1-hydroxypyrene (1-HOP), the most common metabolite of PAHs, which is dependable in assessing tobacco smoke exposure, has been examined in previous studies^21,25^. In our study, the urinary 1-OHP concentrations in non-smoking participants was 0.97 μmol/mol cr, similar to a previous study on staff not exposed to polycyclic aromatic hydrocarbons^43^, but lower than the urinary 1-OHP in non-smoking male service staff in Chinese restaurants^44^. Some studies showed a significantly higher urinary 1-OHP concentration in active smokers, compared with non-smokers^25,26,45^. As expected, our study indicated that urinary 1-OHP concentration was strongly associated with active smoking. In the present study, all the participants worked in one company with similar schedules, and most of them lived in company residential areas and dined in the factory canteen, which could weaken interference from other influencing factors (e.g. cooking, burning coal, domestic air heating, and traffic congestion). In addition, we found that the urinary 1-OHP concentrations had no significant differences between passive smoking and non-smoking, which is consistent with the results of some studies^46^. However, some other studies demonstrated that the urinary 1-OHP concentrations were significantly correlated with passive smoking^21,47^. Possible reasons for this distinction might be that some confounding variables (e.g. traffic congestion, diet, cooking oil fumes exposure) were not taken into consideration in our study and the characteristics of the study populations were significantly different. For instance, the urinary 1-OHP concentration tended to be higher in the populations in industrial regions^48^, heavily trafficked urban areas and among the frequent eaters of grilled meat^46^.

We also found that high concentration of urinary 1-OHP was significantly associated with daytime dysfunction and overall poor sleep quality, compared with the low concentration. The finding is supported by another publication of our team, which surveyed 2197 participants to assess the association between Chinese cooking oil fumes and sleep quality^27^. The biomarker 1-OHP was found to be associated with adult depression^49^, which might affect sleep quality. However, there was no association between urinary 1-OHP levels and short sleep duration in the present study. The possible reasons for the different findings might be due to the fact that female workers were not recruited in our study, while smoking may affect women’s sleep quality more than men’s^6^. Additionally, compared with men, women were more exposed to cooking oil fumes, which could cause poorer sleep quality^27^. Further, studies should focus on the functional mechanism of 1-OHP that generates poor sleep quality.

Our study has several limitations. First, the study design was cross-sectional and thus a causal relationship of active/passive smoking and 1-OHP with poor sleep quality cannot be confirmed. Second, all the participants were middle-aged male workers from one company. Of a total 1787 participants, 1226 (68.6%) did not offer the urine sample on schedule. The non-responding workers might have had more sleep problems related to 1-OHP and/or smoking. Third, in this study, some influencing factors (e.g. caffeine intake, occupation, diet, cooking oil fumes exposure, seasonal effects) were not taken into account, which might cause bias. However, most workers in this study lived in company residential areas, dined in the factory canteen and had a similar dietary habit. In addition, some previous studies have indicated that active smoking and passive smoking were significantly associated with poor sleep quality^6,18,20^. Fourth, several compounds, including exhaled carbon monoxide, hair nicotine, saliva cotinine, and urine cotinine could be used as internal markers for assessing tobacco smoke exposure, but we only measured urinary 1-OHP as the internal exposure marker. Fifth, data on sleep quality were obtained by a self-reported questionnaire method, which might have biased the results. Nevertheless, the PSQI is a scale approved and widely used to evaluate participants’ sleep quality.

## CONCLUSIONS

This study found that both active smoking and passive smoking were significantly associated with long sleep latency, short sleep duration, daytime dysfunction, and overall poor sleep quality among male enterprise workers. Urinary 1-OHP concentration in active smokers was significantly higher than that in non-smokers, and high concentration of urinary 1-OHP was significantly associated with daytime dysfunction and overall poor sleep quality.
